# Analysis of related risk factors of lung metastasis after laparoscopic radical hysterectomy of cervical cancer

**DOI:** 10.1097/MD.0000000000024480

**Published:** 2021-05-07

**Authors:** Liu Henglian, Wang Jiajun, Wang Caixia, Lu Gang, Xia Min

**Affiliations:** Department of Gynecology and Obstetrics, Traditional Chinese Medicine Hospital, Chongqing, China.

**Keywords:** cervical cancer, laparoscopic radical hysterectomy, lung metastasis, risk factors

## Abstract

To explore the risk factors of lung metastasis in patients after laparoscopic radical hysterectomy (LRH) of cervical cancer (CC).

The clinical data of CC patients with clinical stage of IA_1_–IIA_2_ diagnosed in our hospital from April 2007 to October 2015 were collected. According to the situation of metastasis, the patients were divided into lung metastasis (n = 73) and non-lung metastasis group (n = 2076). The clinical data were compared between 2 groups, and logistic stepwise regression model was used to analyze the risk factors of lung metastasis in patients with CC after LRH.

The incidence of lung metastasis after LRH of CC was 3.39%, and 67.13% of patients with lung metastases had no obvious clinical symptoms. 15.06% patients had lung metastasis in the first year, 38.35% in the second year, 43.83% in the third year and later. The postoperative lung metastasis of CC was related to tumor diameter (*P* < .001), pathological type (*P* < .001), interstitial invasion depth (*P* < .001), pelvic lymph node metastasis (PLNM, *P* < .001), vascular tumor thrombus (*P* = .011), tumor uterine invasion (*P* = .002), and abnormal preoperative tumor markers (*P* = .015). However, it was not related to age, clinical stage, tumor growth pattern, tumor differentiation, and para-aortic lymph node metastasis (*P* > .05). Logistic regression analysis revealed non-squamous cell carcinoma (*P* = .022), tumor diameter ≥4 cm (*P* = .008), interstitial invasion depth >2/3 (*P* = .003), PLNM (*P* = .007), and tumor uterine invasion (*P* = .037) is an independent risk factor for lung metastasis after LRH of CC.

Non-squamous cell carcinoma, tumor diameter ≥4 cm, tumor interstitial invasion depth >2/3, PLNM, and tumor uterine invasion are independent risk factors for lung metastasis after LRH of CC.

## 1 Introduction

Cervical carcinoma (CC) is a common tumor in women and the number of new cases worldwide shows about 500,000/yr, 80% of which occurred in developing countries.^[1–3]^ At present, CC is seriously threatening the health of women, and the patients are getting younger.^[1–3]^ With the improvement of diagnosis and treatment of CC, the survival rate of CC is significantly increased, but there are still many patients with distant organ metastasis, which seriously affects the physical and mental health of patients.^[4–6]^ Lung is the main metastasis site of CC, and the probability of lung metastasis after laparoscopic radical hysterectomy (LRH) of CC is about 2.1% to 6.1%. Lung metastasis seriously affects the prognosis of patients with CC.^[7–9]^ In current investigation, the clinicopathological data of 2179 patients with CC after LRH were collected retrospectively to explore the independent risk factors of lung metastasis in CC patients, so as to provide reference for the prevention of lung metastasis in CC.

## 2 Materials and methods

### 2.1 Inclusion and exclusion of clinical cases

From April 2007 to October 2015, the clinical data of patients with CC in our hospital were analyzed retrospectively. Inclusion criteria: patients with CC were diagnosed by preoperative cervical biopsy and postoperative pathology; CC patients with clinical stage of IA_1_–IIA_2_; preoperative computed tomography (CT) imaging examination showed no occupying lung disease; the subjects underwent LRH and pelvic lymphadenectomy; patients with complete clinical data and follow-up medical records. Exclusion criteria: patients with other malignancies; patients with incomplete clinical data and lost visit; patients with space occupying lesions in the lung. This study was approved by the Ethics Committee of Chongqing Traditional Chinese Medicine Hospital (registration number: cqzyy2009201512).

### 2.2 Clinical data and research methods

According to the inclusion and exclusion criteria, the data of hospitalization and/or outpatient follow-up of patients included in the study in the past 5 years were collected. The patients were followed up once every 3 months after operation. Routine reexamination items include: ultrasound, chest X-ray, CT, serum tumor marker test, etc. Patients with pulmonary nodules were diagnosed with chest enhanced CT or positron emission computed tomography (PET-CT). According to whether patients had lung metastasis after operation, they were divided into lung metastasis group (n = 73) and non-lung metastasis group (n = 2076). The diagnosis of pulmonary metastasis is mainly based on the results of CT and/or PET-CT. The postoperative adjuvant chemotherapy was Nedaplatin combined with paclitaxel, and the radiotherapy was 6mvx for three-dimensional conformal radiotherapy (3D-CRT), DT: 46–48 Gy. The age, clinical stage, tumor growth mode, tumor diameter, pathological type, interstitial invasion depth, vascular tumor thrombus, pelvic lymph node metastasis (PLNM), tumor uterine invasion, para-aortic lymph node metastasis, abnormal preoperative tumor markers, and other clinical data were collected for analysis. Abnormal serum tumor markers refer to serum squamous cell carcinoma antigens in patients with squamous cell carcinoma ≥1.5 μg/L, serum carbohydrate antigens 125 in patients with adenocarcinoma ≥35U/mL, and/or carbohydrate antigens 19–9 ≥37 U/mL, serum neuron-specific enolase of patients with neuroendocrine cancer ≥12.5 μg/L.

### 2.3 Statistical analysis

All data were analyzed by SPSS 19.0 software (Tokyo, Japan). Categorical variables were described by frequency and compared by chi-square test or Fisher test. Logistic stepwise regression model was used to analyze the independent risk factors of lung metastasis in patients with CC after LRH. The odds ratio (OR) and 95% confidence interval (95% CI) were calculated. *P* < .05 was considered statistically significant.

## 3 Result

### 3.1 Analysis of clinical symptoms and metastasis in patients with lung metastasis

According to the inclusion and exclusion criteria, a total of 2149 patients were included in the study. Seventy three patients with and 2076 patients without pulmonary metastasis were enrolled for investigation. The incidence of pulmonary metastasis was 3.39%. In the lung metastasis group, 24 patients (32.87%) had cough, expectoration, chest distress, and other clinical symptoms. The other 49 patients (67.13%) without obvious symptoms were found in the chest imaging examination and/or abnormal tumor markers in serum. In the lung metastasis group, 11 patients (15.06%) had lung metastasis in the first year, 30 (38.35%) in the second year, and 32 (43.83%) in the third year and later. Also, 36 cases (49.32%) had double lung metastasis, 17 cases (23.29%) had right lung metastasis, and 20 cases (27.39%) had left lung metastasis.

### 3.2 Comparison of clinical data between the 2 groups

As shown in Table 1, the univariate analysis of clinical data of patients with lung metastasis group and patients without lung metastasis found that the postoperative lung metastasis of CC was related to tumor diameter (*P* < .001), pathological type (*P* < .001), interstitial invasion depth (*P* < .001), PLNM (*P* < .001), vascular tumor thrombus (*P* = .011), tumor uterine invasion (*P* = .002), and abnormal preoperative tumor markers (*P* = .015). However, it was not related to age, clinical stage, tumor growth pattern, tumor differentiation, and para-aortic lymph node metastasis (*P* > .05).

**Table 1:** Comparison of clinical data between the 2 groups.

Parameter	Lung metastasis group (n = 73)	Non-lung metastasis group (n = 2076)	*P* value
Age, y			
≤40	15 (20.54%)	396 (19.07%)	
>40	58 (79.45%)	2072 (80.93%)	.765
Clinical stages			
IA1–IA2	5 (6.86%)	160 (7.71%)	
IB1–IB2	40 (52.35%)	1112 (53.56%)	
IIA1–IIA2	28 (38.39%)	804 (38.72%)	.189
Pathological type			
Squamous cell carcinoma	55 (75.34%)	1804 (86.89%)	
Non-squamous cell carcinoma	18 (24.66%)	272 (13.10%)	<.001
Tumor growth pattern			
Exogenous type	25 (34.25%)	780 (37.57%)	
Endophytic type	48 (65.75%)	1296 (62.43%)	.582
Tumor differentiation			
Highly differentiated	8 (10.96%)	232 (11.17%)	
Moderately differentiated	43 (58.90%)	1284 (61.85%)	
Poorly differentiated	22 (30.14%)	560 (26.97%)	.849
Tumor diameter, cm			
≥4	45 (61.64%)	512 (24.67%)	
<4	28 (38.36%)	1564 (75.33%)	<.001
Interstitial invasion depth			
<1/3	18 (24.65%)	1100 (52.99%)	
1/3–2/3	25 (34.25%)	640 (30.83%)	
>2/3	30 (41.10%)	336 (16.18%)	<.001
Pelvic lymph node metastasis			
Yes	29 (39.73%)	1536 (73.99%)	
No	44 (60.27%)	540 (26.01%)	<.001
Para-aortic lymph node metastasis			
Yes	66 (90.41%)	1928 (92.87%)	
No	7 (9.59%)	148 (7.13%)	.453
Vascular tumor thrombus			
Yes	48 (69.86%)	1032 (49.71%)	
No	25 (30.14%)	1044 (20.29%)	.011
Tumor uterine invasion			
Yes	23 (31.51%)	244 (11.75%)	
No	50 (68.49%)	1832 (88.25%)	.002
Abnormal preoperative tumor markers			
Normal	26 (35.62%)	1056 (50.87%)	
Abnormal	47 (64.38%)	1020 (49.13%)	.015

### 3.3 Analysis of independent risk factors for pulmonary metastasis after LRH of CC

As shown in Table 2, the logistic regression analysis of clinical data of patients with lung metastasis group and patients without lung metastasis found that non-squamous cell carcinoma (OR = 2.942, 95% CI = 2.007–7.981, *P* = .022), tumor diameter ≥4 cm (OR = 3.285, 95% CI = 2.248–6.374, *P* = .008), interstitial invasion depth >2/3 (OR = 1.842, 95% CI = 1.164–3.867, *P* = .003), PLNM (OR = 3.456, 95% CI = 2.268–8.691, *P* = .007), and tumor uterine invasion (OR = 2.814, 95% CI = 1.901–5.039, *P* = .037) were independent risk factor for lung metastasis after LRH of CC.

**Table 2:** Analysis of independent risk factors for pulmonary metastasis after laparoscopic radical hysterectomy of early stage cervical cancer.

Parameter	OR value	95% CI	*P* value
Pathological type	2.942	2.007–7.981	.022
Tumor diameter, cm	3.285	2.248–6.374	.008
Interstitial invasion depth	1.842	1.164–3.867	.003
Pelvic lymph node metastasis	3.456	2.268–8.691	.007
Vascular tumor thrombus	2.693	1.101–3.254	.211
Tumor uterine invasion	2.814	1.901–5.039	.037
Abnormal preoperative tumor markers	2.737	0.945–3.012	.173

## 4 Discussion

In this study, we found the incidence of lung metastasis after LRH of CC was 3.39% and non-squamous cell carcinoma, tumor diameter ≥4 cm, tumor interstitial invasion depth >2/3, PLNM, and tumor uterine invasion are independent risk factors for lung metastasis after LRH of CC. Early identification of risk factors for lung metastasis after LRH of CC has important clinical value for the prevention and predicting the prognosis of CC.

In recent years, with the promotion of cytological screening, more potential CC patients have been diagnosed; on the other hand, with the improvement of modern medical technology, the treatment methods of CC continue to improve, and the mortality rate of patients has decreased significantly, but some CC patients still have distant metastasis, and the lung is the main organ of distant metastasis.^[7,8,9,10,11]^ The incidence of lung metastasis in CC was 2.1% to 6.1% reported in previous literature. Lung metastasis seriously affected the survival rate and prognosis of CC patients.^[7,12]^ In this work, we discovered that the incidence of lung metastasis after LRH of CC was 3.39%. 67.13% of patients with lung metastasis had no obvious symptoms, which was often found due to abnormal chest imaging and (or) serum tumor markers.

More and more scholars have realized the influence of tumor diameter on the survival rate of CC patients.^[13,14]^ Nakanish et al^[14]^ analyzed 566 cases of CC and found that tumor diameter >4 cm can be used as a threshold for poor prognosis. In this work, we found that tumor diameter >4 cm was an independent risk factor for lung metastasis after LRH of CC. The risk of lung metastasis in patients with tumor diameter >4 cm was 3.285 times (95% CI: 2.248–6.374) higher than that in patients with tumor diameter ≤4 cm. Therefore, for the CC patients whose tumor diameter is >4 cm, the choice of operation method and time should be cautious. Preoperative and postoperative adjuvant chemotherapy can be considered to reduce tumor cell viability, inactivate small metastatic lesions, reduces residual lesions during surgery and postoperative lung metastases. Similar to tumor diameter, the deeper the tumor infiltrate, the more likely the lung metastasis will occur. The results of multivariate analysis suggested that the depth of interstitial infiltration >2/3 was an independent risk factor for lung metastasis, which was similar to the conclusions of Kristensen et al^[15]^ and Ho et al.^[16]^

PLNM is a relatively recognized adverse factor affecting the prognosis of patients with CC. Although whether PLNM does not change the clinical stage of CC, it is one of the most important independent risk factors for poor prognosis and recurrence of CC.^[17]^ Previous studies suggest that PLNM is an independent risk factor for lung metastasis after radical surgery for CC.^[18]^ In this work, we also found that the risk of lung metastasis in patients with PLNM is 3.456 times (95% CI = 2.268–8.691) higher than that in patients without PLNM. Literature reports suggest that there is a close relationship between tumor uterine invasion and PLNM in patients with CC. The rate of PLNM in CC patients with uterine infiltration is significantly increased, and the prognosis of patients is poor.^[19]^ In this study, we found that positive uterine invasion was an independent risk factor for lung metastasis in patients with LRH of CC. Patients with positive uterine invasion were 2.814 times more likely to develop lung metastasis than patients without positive uterine invasion (95% CI = 1.901–5.039). The influence of pathological types on the prognosis of CC is still controversial. In CC, research suggests that non-squamous cancer patients have a worse prognosis than squamous cancer patients.^[20–23]^ Yamauchi analyzed 455 patients found that the prognosis of patients with adenocarcinoma is worse than that of patients with squamous cancer, especially in patients with stage I.^[21]^ In this study, Logistic regression analysis showed that lung metastasis in patients with non-squamous carcinoma was 2.954 times that in patients with squamous carcinoma (OR = 2.942, 95% CI = 2.007–7.981). The possible reason is that squamous cell carcinoma is mostly local infiltrative growth, with less distant metastasis in the early stage, while adenocarcinoma is more likely to have lymphatic and hematogenous metastasis, so non squamous cell carcinoma is more likely to have lung metastasis.^[24,25]^ In addition, in this study, univariate analysis found that abnormal serum tumor markers and vascular tumor thrombus were related to lung metastasis after LRH of CC, but there was no statistical significance in logistic regression analysis (*P* > .05). This may be because the effects of the above factors on lung metastasis are masked by stronger factors, or because the study sample size is small and the statistical results are biased.

In this way, this research has certain limitations. First, the research sample is small, and the research results need to be further confirmed by a large prospective sample. Second, this research is a single-center regression study, the research results can only reflect the situation of the unit and future multicenter research needs to be further developed. Third, due to the characteristics of surgical proficiency and population differences, the results of this study may have inherent errors. Fourth, this research has all the limitations and risks of bias inherent in research design.

## Author contributions

**Conceptualization:** Liu Henglian, Xia min, Wang Caixia, Lu Gang.

**Data curation:** Liu Henglian, Xia min, Wang Jiajun, Wang Caixia, Lu Gang.

**Formal analysis:** Liu Henglian, Wang Jiajun, Wang Caixia, Lu Gang.

**Funding acquisition:** Liu Henglian, Xia min, Wang Jiajun.

**Investigation:** Wang Jiajun, Wang Caixia, Lu Gang.

**Methodology:** Liu Henglian, Xia min.

**Resources:** Liu Henglian, Xia min, Wang Jiajun, Wang Caixia, Lu Gang.

**Software:** Xia min, Wang Jiajun.

**Supervision:** Liu Henglian.

**Validation:** Liu Henglian, Xia min, Wang Jiajun, Wang Caixia, Lu Gang.

**Visualization:** Liu Henglian, Xia min.

**Writing – original draft:** Liu Henglian, Xia min, Wang Jiajun.

**Writing – review & editing:** Xia min, Wang Jiajun, Wang Caixia.

## References

[1] Waggoner SE. Cervial cancer. Lancet 2003;361:2217–25. 12842378 10.1016/S0140-6736(03)13778-6

[2] Zeng H Chen W Zheng R. Changing cancer survival in China during 2003–15: a pooled analysis of 17 population-based cancer registries. Lancet Glob Health 2018;6:e555–67. 29653628 10.1016/S2214-109X(18)30127-X

[3] Shrestha AD Neupane D Vedsted P. CC prevalence, incidence and mortality in low and middle income countries: a systematic review. Asian Pac J Cancer Prev 2018;19:319–24. 29479954 10.22034/APJCP.2018.19.2.319 PMC5980914

[4] Cui L Shi Y Zhang GN. Perineural invasion as a prognostic factor for CC: a systematic review and meta-analysis. Arch Gynecol Obstet 2015;292:13–9. 25637504 10.1007/s00404-015-3627-z

[5] Liontos M Kyriazoglou A Dimitriadis I. Systemic therapy in CC: 30 years in review. Crit Rev Oncol Hematol 2019;137:09–17. 10.1016/j.critrevonc.2019.02.009 31014518

[6] Bentivegna E Gouy S Maulard A. Oncological outcomes after fertility-sparing surgery for CC: a systematic review. Lancet Oncol 2016;17:e240–53. 27299280 10.1016/S1470-2045(16)30032-8

[7] Ki EY Lee KH Park JS. A clinicopathological review of pulmonary metastasis from uterine CC. Cancer Res Treat 2016;48:266–72. 25715766 10.4143/crt.2014.206 PMC4720087

[8] Li H Wu X Cheng X. Advances in diagnosis and treatment of metastatic CC. J Gynecol Oncol 2016;27:e43. 27171673 10.3802/jgo.2016.27.e43 PMC4864519

[9] Suffredini DA Lee JM Peer CJ. Pulmonary tumor thrombotic microangiopathy and pulmonary veno-occlusive disease in a woman with CC treated with cediranib and durvalumab. BMC Pulm Med 2018;18:112. 29996818 10.1186/s12890-018-0681-x PMC6042377

[10] Burki TK. Novel mutations in CC. Lancet Oncol 2017;18:e137. 28163002 10.1016/S1470-2045(17)30080-3

[11] Denny L. Prevention and treatment. Discov Med 2012;14:125–31. 22935209

[12] Kanthan R Senger JL Diudea D. Pulmonry lymphangitic carcinomatosis from squamous cell carcinoma of the cervix. World J Surg Oncol 2010;8:107. 21129213 10.1186/1477-7819-8-107 PMC3006383

[13] Xie XZ Song K Cui B. Clinical and pathological factors related to the prognosis of chinese patients with stage Ib to IIb CC. Asian Pac J Cancer Prev 2012;13:5505–10. 23317208 10.7314/apjcp.2012.13.11.5505

[14] Nakanishi T Ishikawa H Nawa A. The significance of tumor size in clinical stage IB CC: Can a cut-off figure be determined? Int J Gynecol Cancer 2000;10:397–401. 11240704 10.1046/j.1525-1438.2000.010005397.x

[15] Kristensen GB Abeler VM Risberg B. Tumor size, depth of invasion, and grading of the invasive tumor front are the main prognostic factors in early squamous cell cervical carcinoma. Gynecol Oncol 1999;74:245–51. 10419739 10.1006/gyno.1999.5420

[16] Ho CM Chien TY Huang SH. Multivariate analysis of the prognostic factors and outcomes in early CC patientsundergoing radical hysterectomy. Gynecol Oncol 2004;93:458–64. 15099962 10.1016/j.ygyno.2004.01.026

[17] Hu T Li X Zhang Q. Could the extent of lymphadenectomy be modified by neoadjuvant chemotherapy in cervicalcancer? A large-scale retrospective study. PLoS One 2015;10:e0123539. 25859857 10.1371/journal.pone.0123539 PMC4393094

[18] Zheng A Chen Y Fang J. Clinicopathologic characteristics and risk factors for lung metastasis after radical hysterectomy in early-stage CC. Zhonghua Fu Chan Ke Za Zhi 2015;50:204–9. 26268411

[19] Jouglar E Thomas L Mahé MA. Toxicity and early clinical outcomes in CC following extended field helical tomotherapy to para-aortic lymph nodes. Cancer Radiother 2016;20:794–800. 28270323 10.1016/j.canrad.2016.06.007

[20] Xie X Song K Cui B. A comparison of the prognosis between adenocarcinoma and squamous cell carcinoma in stage IB-IIA CC. Int J Clin Oncol 2018;23:522–31. 29299705 10.1007/s10147-017-1225-8

[21] Yamauchi M Fukuda T Wada T. Comparison of outcomes between squamous cell carcinoma and adenocarcinoma in patientswith surgically treated stage I-II CC. Mol Clin Oncol 2014;2:518–24. 24940487 10.3892/mco.2014.295 PMC4051565

[22] Canaz E Ozyurek ES Erdem B. Preoperatively assessable clinical and pathological risk factors for parametrial involvement in surgically treated FIGO Stage IB-IIA CC. Int J Gynecol Cancer 2017;27:1722–8. 28617687 10.1097/IGC.0000000000001060

[23] Zhu T Chen X Zhu J. Surgical and pathological outcomes of laparoscopic versus abdominal radical hysterectomy with pelvic lymphadenectomy and/or para-aortic lymph node sampling for bulky early-stage CC. Int J Gynecol Cancer 2017;27:1222–7. 28640767 10.1097/IGC.0000000000000716

[24] Charakorn C Thadanipon K Chaijindaratana S. The association between serum squamous cell carcinoma antigen and recurrence and survival of patients with cervical squamous cell carcinoma: a systematic review and meta-analysis. Gynecol Oncol 2018;150:190–200. 29606483 10.1016/j.ygyno.2018.03.056

[25] Waggoner SE. Cervical cancer. Lancet 2003;361:2217–25. 12842378 10.1016/S0140-6736(03)13778-6

